# Renin angiotensin system molecules and chemokine (C-C motif) ligand 2 (CCL2) in chronic kidney disease patients

**DOI:** 10.1590/2175-8239-JBN-2021-0030

**Published:** 2021-07-07

**Authors:** Isabella Viana Gomes Schettini, Débora Vargas Faria, Leilismara Sousa Nogueira, Alba Otoni, Ana Cristina Simões e Silva, Danyelle Romana Alves Rios

**Affiliations:** 1 Universidade Federal de São João del-Rei, Campus Centro Oeste, Divinópolis, MG, Brasil.; 2 Universidade Federal de Minas Gerais, Faculdade de Medicina, Laboratório Interdisciplinar de Investigação Médica, Departamento de Pediatria, Belo Horizonte, MG, Brasil.

**Keywords:** Renal Insufficiency, Chronic, Renin-Angiotensin System, CCL2, Insuficiência Renal Crônica, Sistema Renina-Angiotensina, CCL2

## Abstract

**Introduction:**

Studies have shown that the renin angiotensin aldosterone system (RAAS) and inflammation are related to kidney injury progression. The aim of this study was to evaluate RAAS molecules and chemokine (C-C motif) ligand 2 (CCL2) in 82 patients with chronic kidney disease (CKD).

**Methods:**

Patients were divided into two groups: patients diagnosed with CKD and patients without a CKD diagnosis. Glomerular filtration rate (GFR) and albumin/creatinine ratio (ACR) were determined, as well as plasma levels of angiotensin-(1-7) [Ang-(1-7)], angiotensin-converting enzyme (ACE)1, ACE2, and plasma and urinary levels of CCL2.

**Results:**

CCL2 plasma levels were significantly higher in patients with CKD compared to the control group. Patients with lower GFR had higher plasma levels of ACE2 and CCL2 and lower ratio ACE1/ACE2. Patients with higher ACR values had higher ACE1 plasma levels.

**Conclusion:**

Patients with CKD showed greater activity of both RAAS axes, the classic and alternative, and higher plasma levels of CCL2. Therefore, plasma levels of RAAS molecules and CCL2 seem to be promising prognostic markers and even therapeutic targets for CKD.

## Resumo

**Introdução:**

Estudos têm mostrado que o sistema renina angiotensina aldosterona (SRAA) e a inflamação estão relacionados à progressão da lesão renal. O objetivo deste estudo foi avaliar moléculas do SRAA e o Ligante 2 de Quimiocina com Motivo C-C (CCL2) em 82 pacientes com doença renal crônica (DRC).

**Métodos:**

Os pacientes foram divididos em dois grupos: pacientes diagnosticados com DRC e pacientes sem diagnóstico de DRC. Foram determinadas a taxa de filtração glomerular (TFG) e a relação albumina/creatinina (RAC), assim como os níveis plasmáticos de angiotensina-(1-7) [Ang-(1-7)], enzima conversora de angiotensina (ECA)1, ECA2 e níveis plasmáticos e urinários de CCL2.

**Resultados:**

Os níveis plasmáticos de CCL2 foram significativamente mais altos em pacientes com DRC em comparação com o grupo controle. Pacientes com TFG mais baixa apresentaram níveis plasmáticos mais elevados de ECA2 e CCL2 e menor relação ECA1/ECA2. Pacientes com valores de RAC mais altos apresentaram níveis plasmáticos de ECA1 mais elevados.

**Conclusão:**

Pacientes com DRC mostraram maior atividade de ambos os eixos do SRAA, o clássico e o alternativo, e níveis plasmáticos mais altos de CCL2. Portanto, os níveis plasmáticos de moléculas do SRAA e CCL2 parecem ser marcadores prognósticos promissores e até mesmo alvos terapêuticos para a DRC.

## Introduction

The Kidney Disease Improving Global Outcomes (KDIGO) defines chronic kidney disease (CKD) as functional or structural abnormalities of kidneys, persisting for more than three months and with health implications for the patient. The assessment of kidney impairment is recommended by assessing albuminuria, mainly as spot urine albumin/creatinine ratio (ACR), and by assessing renal function with the estimated glomerular filtration rate (GFR) based on serum creatinine values or available equations^1^. CKD is considered a major public health problem worldwide, with an estimated prevalence of up to 15%^2^ causing a great negative impact on the life expectancy and quality of the patients and demanding a significant part of resources allocated to health^3^. In Brazil, it is estimated that more than ten million people have the disease^4^. Of these, about 130 thousand undergo dialysis therapy and 82% of dialysis performed in the country is financed by the Public Health System (SUS) 4 ^,^ 5, with an estimated annual expenditure of more than R$ 2 billion^6^.

In Brazil, North America, and countries in the European continent, diabetes mellitus, hypertension, and glomerular diseases are the main causes of CKD. The main mechanisms of arterial hypertension in CKD are saline and volume overload, in addition to increased activity of renin angiotensin aldosterone system (RAAS), endothelial dysfunction, and inflammation^1^.

RAAS is considered an endocrine system, formed by peptides, enzymes, and receptors, responsible for cardiovascular, renal, and adrenal regulation, which indirectly controls the balance of fluids and electrolytes and actively participates in the regulation of vasomotor tone and cell proliferation. Effects of RAAS can widely affect functions and renal diseases through multiple mediators and receptors 7 ^,^ 8. Currently, it is considered a system formed by two opposite axes: 1) classic axis, initiated by the cleavage of angiotensinogen into angiotensin I (Ang I), by the action of renin, and which is later converted on angiotensin II (Ang II), by the angiotensin-converting enzyme 1 (ACE1); and 2) alternative or counter-regulatory axis, through the cleavage of Ang II and consequent production of heptapeptide angiotensin-(1-7) [Ang-(1-7)] by action of an enzyme homologous to ACE, the angiotensin-converting enzyme 2 (ACE2)^9^ .

RAAS is closely associated with mechanisms of kidney injury progression. Experimental studies have shown that Ang II participates in renal hemodynamic changes and inflammatory and fibrotic processes responsible for progression of kidney damage^7^^,^ 10. Inhibition of formation and/or binding of Ang II to its AT1 receptor (ARA) can slow the progression of renal fibrosis and reduce mortality and the risk of cardiovascular complications related to CKD^10^^,^ 11.

Inflammation also plays an important role in the development of CKD, participating actively in harmful mechanisms by activating the immune system in a continuous and exacerbated manner. Studies on inflammatory mediators have indicated an important role for chemokines in CKD^8^^,^ 12. The monocyte-1 chemoattractant protein (MCP-1), also known as chemokine (C-C motif) ligand 2 (CCL2) has been reported as an important inflammatory mediator of renal diseases, being expressed in practically all types of intrinsic renal cells (endothelial, mesangial, and tubular epithelial cells and podocytes) in the presence of renal tissue damage^13^.

In this regard, this study aimed to evaluate the association between plasma levels of the RAAS molecules and plasma and urinary levels of CCL2 with CKD markers in patients with and without CKD. 

## Materials and Methods

### Study design and population

This was a cross-sectional study conducted with patients exhibiting arterial hypertension and/or diabetes mellitus, registered in the Hiperdia Program of a medium-sized municipality in the state of Minas Gerais, as previously reported elsewhere 14. Briefly, patients were randomly selected from a group of hypertensive individuals at high risk for cardiovascular disease, according to the revised Framingham score, adopted by Minas Gerais State Health Secretariat, and patients with diabetes. The 82 patients included in this study were divided into two groups: (1) patients diagnosed with CKD (CKD group), who showed changes in markers of renal function and (2) patients without diagnosis of CKD randomly chosen (control group).

#### Data And Biological Sample Collection

Data collection, referring to age and sex of patients, was performed directly from the Hiperdia program in the municipality. Samples of 10 mL of venous blood were collected from the antecubital region in dry and EDTA tubes of the Vacutainer system (Becton Dickinson) after a 10-12h fasting period. A sample of first morning urine was also collected in a sterile flask by patient himself. Blood samples were centrifuged at 3500 rpm for 15 minutes in a CentriBio^®^ centrifuge to obtain serum and plasma. These were aliquoted in Eppendorf^®^ tubes and stored at -80ºC together with urine aliquots, properly homogenized, until the time of measurements.

### Study Variables

#### Response Variable: Plasma Levels Of Ang-(1-7), Ace1, And Ace2 And Plasma And Urinary Ccl2 Levels

Measurements of plasma protein levels of Ang-(1-7), ACE1, ACE2, and plasma and urinary CCL2 levels were performed by quantitative ELISA, using MyBioSource kits: Human Angiotensin 1-7 (ANG1-7), Human Angiotensin I Converting Enzyme (ACE1), Human Angiotensin II Converting Enzyme (ACE2) ELISA kits, and R&D Systems kits Quantikine^®^ ELISA Human MCP-1/CCL2, respectively, following the manufacturer’s instructions. Briefly, these kits apply the enzyme immunoassay technique utilizing monoclonal antibodies, antigen-HRP conjugate, and substrate for HRP enzyme, which form a colored complex. The color intensity is measured spectrophotometrically at 450 nm in a microplate reader. A standard curve is plotted relating the intensity of the color to the concentration of standards. The analytes’ concentration in each sample is interpolated from this standard curve.

The variable “ACE1/ACE2 ratio” was obtained by dividing the continuous variables ACE1 and ACE2.

### Explanatory Variables: Ckd Diagnosis

CKD diagnosis (stage 3 or higher) was defined by GFR < 60mL/min/1.73m^2^ and/or ACR≥ 30 mg/g in two consecutive evaluations with an interval of three months. Serum creatinine dosage was determined by colorimetric kinetic test, using K067 Kit from Bioclin^®^. The GFR was estimated by the Chronic Kidney Disease Epidemiology Collaboration (CKD-EPI) equation and categorized in GFR <60 mL/min/1.73 m^2^ or GFR ≥60 mL/min/1.73 m^2^. Albuminuria was measured using a turbidimetric method with the *K078* Kit from Bioclin^®^. ACR was calculated from the measurements of albumin and creatinine in urine samples, and was categorized into ACR ≥30 and ACR <30.

### Statistical analysis

Continuous variables were described using median and interquartile range (IQR) or mean and standard deviation, and categorical variables using proportions. The normality of data was assessed by histogram analysis. Categorical variables were compared using the chi-square test and continuous variables by the *t* test or Mann-Whitney test. The correlation between GFR and ACR (continuous variables) with plasma levels of Angiotensin (1-7), ACE1, and ACE2 and plasma and urinary CCL2 levels was assessed using Spearman correlation test. Significant differences were considered for p values < 0.05 and statistical analyses were performed using STATA software (version 14.0) for Windows.

### Ethical aspects

The study was previously approved by the Research Ethics Committee of the Federal University of São Joao Del-Rei, CAAE: 30836414.1.0000.5545. All patients signed the informed consent form.

## Results

The majority of patients in both groups were male and the average age of patients with CKD was significantly higher when compared to the control group. The mean GFR and median ACR for patients with CKD were 50.0±16.3 mL/min/1.73m^2^ and 18.6 (2.5-86.3) and of the control group, 74.8±9.7 and 2.6 (1.0-5.3), respectively. 

Plasma levels of Ang 1-7, ACE1, ACE2 and urinary levels of CCL2 were not statistically different between CKD and control groups. Only plasma levels of CCL2 were significantly higher in patients with CKD when compared to the control group (Table 1).

**Table 1:** Comparison of sociodemographic and RAAS markers between CKD and control groups (N = 82)

Variable	CKD group (n=41)	Control group (n=41)	p value
Age (years)	64.6 ± 11.0	53.9 ± 13.6	<0.01*
Gender [n(%)]			0.824
Male	19 (46%)	18 (44%)	
Female	22 (54%)	23 (56%)	
Plasma Ang 1-7 (pg/mL)	68.8 (23.3-179.9)	100.1 (25.9-212.0)	0.74
Plasma ACE1(pg/mL)	58.7 ± 25.3	53.1 ± 21.8	0.29
Plasma ACE2 (pg/mL)	16.4 (0.0-43.3)	4.2 (0.0-25.5)	0.26
Plasma CCL2 (pg/mL)	148.7 (114.6-187. 5)	116.7 (92.0-145.0)	<0.05*
Urinary CCL2 (pg/mL)	225.3 (145.3-420.4)	175.5 (71.8-295.2)	0.17
ACE1/ACE2	1.8 (1.2-4.4)	2.5 (0.9-9.4)	0.44

Mean ± SD or median (25% -75%). GFR: Glomerular Filtration Rate. ACR: Albumin-creatinine ratio. Ang-1-7: Angiotensin 1-7. ACE1: Angiotensin-converting

Patients with lower GFR had higher plasma levels of ACE2 and CCL2 and a lower ACE1/ACE2 ratio. Patients with higher ACR values had higher ACE1 plasma levels. There was no statistically significant correlation between GFR and ACR and the other evaluated markers (Table 2).

**Table 2:** Correlation between GFR and ACR with plasma levels of Angiotensin 1-7, ACE1, ACE2, and plasma and urinary CCL2 levels

Variables	Correlation Coefficient	p value
GFR x Ang 1-7	-0.01	0.88
GFR x ACE1	-0.05	0.64
GFR x ACE2	-0.31	<0.01*
GFR x plasma CCL2	-0.32	<0.01*
GFR x urinary CCL2	-0.08	0.54
GFR x ACE1/ACE2	0.33	<0.01*
ACR x Ang 1-7	-0.18	0.10
ACR x ACE1	0.27	<0.01*
ACR x ACE2	0.06	0.53
ACR x plasma CCL2	-0.07	0.59
ACR x urinary CCL2	0.12	0.35
ACR x ACE1/ACE2	-0.19	0.15

GFR: Glomerular filtration rate. ACR: Albumin-creatinine ratio. Ang-1-7: Angiotensin 1-7. ACE1: Angiotensin-converting enzyme 1. ACE2: Angiotensin-converting enzyme 2. CCL2: chemokine (C-C motif) ligand 2.

* p <0.05.

## Discussion

Our study aimed to evaluate the components of the RAAS, plasma and urinary levels of CLL2, and markers of CKD. Plasma CCL2 levels were significantly higher in patients with CKD compared to the control group and a statistically significant negative correlation was found between GFR and ACE2 plasma levels (rho=-0.31) and CCL2 (rho= -0.32) and statistically significant positive correlation between GFR and ACE1/ACE2 (rho= 0.33) and between ACR and ACE1 plasma levels (rho= 0.27).

CCL2 is a chemokine of the CC family, which recruits cells from the monocyte-macrophage lineage, stimulates the release of histamine by basophils, and acts both at early stages and during the progression of renal tubule-interstitial injury^8^, being considered a critical mediator of kidney injury^15^^,^ 16. Studies have shown that this chemokine is present in large quantities in kidneys of patients with glomerular diseases, transplant rejections, interstitial nephritis, or even only in the presence of proteinuria 17 ^,^ 18. In addition, pharmacological inhibition of CCL2 was able to reduce inflammation and improve podocyte function in diabetic nephropathy 19, as well as recover kidney function in patients with diabetes mellitus who experienced albuminuria^20^^,^ 21. 

Several studies have assessed the association between urinary and plasma CCL2 levels and CKD of different etiologies. Rovin *et al.* showed that CCL2 was present in the urine of patients with glomerular diseases. In addition, patients with inflammatory glomerulopathies had higher levels of urinary CLL2 that was directly related to proteinuria. Another relevant data from that study was that the urinary CCL2 was still biologically active, since, in a micro-chemotaxis trial, monocyte migration was increased 22. In diabetic nephropathy, urinary CCL2 was directly related to the risk of CKD progression in patients with macroalbuminuria. However, plasma CCL2 showed no difference in this scenario^23^. 

In our study, plasma CCL2 levels were higher among patients with CKD, confirmed by the association between decreased GFR and increased plasma CCL2. On the other hand, there was no difference in relation to urinary CCL2 levels. Other studies have also identified an inversely proportional correlation between urinary CCL2^24^ or plasma CCL2 25 ^,^ 26 and GFR. Although our study showed a high concentration of plasma CCL2, it is not always high in CKD (27, 28). Urinary CCL2 levels are often higher in these patients, especially in cases when the tubulointerstitial lesions are more advanced 27 ^,^ 28. As an example, in the study by Rovin *et al*, urinary CCL2 did not associate with plasma CCL2, but higher levels were detected in patients with severe glomerular lesions than in those with less severe lesions^22^. The failure to observe a difference in the urinary concentration of CCL2 can be justified by the stage of CKD of the patients included in this study. According to KDIGO^1^, the range of GFR used in this study includes the categories “Mildly decreased” to “Moderately to severely decreased”. Regarding the classification according to the ACR, patients are included in the “Normal to mildly increased” and “Moderately increased” ranges.

It is worth mentioning that there is still no conclusive evidence if the increase in plasma levels of these chemokines is related to the increase in their production or exclusively to the decrease in their clearance, due to their molecular weight (29). Additionally, although CCL2 has been shown to be a relevant marker of CKD, reference values for plasma and urinary chemokines as early immunological biomarkers of kidney injury in patients with glomerulopathies of different causes are not yet available in the literature.

Vianna *et al.*(2011)^8^ suggested that the increase in plasma levels of pro-inflammatory cytokines in patients with CKD may be caused by reduced kidney function, volume overload, oxidative stress, decreased antioxidant levels, and increased RAAS activity. 

Our study showed that patients with lower GFR values had higher plasma levels of ACE2 (p<0.01). These results corroborate the study by Roberts *et al.* 2013)^30^ showing that patients with CKD, at pre-dialysis stage, or kidney transplant patients had elevated ACE2 levels if compared to those undergoing hemodialysis. As an explanation, the authors suggested that ACE2 circulation may increase early in the course of CKD and can be followed by a relative decrease. It is worth mentioning that none of the patients in our study was in the dialysis phase. Furthermore, the study by Soro-Paavonen *et al.* (2012)^31^ also reported that patients with type 1 diabetes and microalbuminuria had elevated ACE2 levels when compared to those who did not have microalbuminuria. 

ACE2 belongs to the so-called counter-regulatory RAAS axis formed by ACE2/Angiotensin-(1-7) [Ang-(1-7)]/Mas receptor. ACE2 converts Ang II into Ang-(1-7), which is responsible for mediating important kidney actions^32^^,^ 33, acting via the Mas receptor. The binding of Ang-(1-7) to Mas receptor leads to vasodilation and antihypertensive, anti-inflammatory, and antifibrotic effects (12). Both ACE2 and Ang-(1-7) can influence the RAAS in the kidneys, by modulating or even counterbalancing the over activity of the classic RAAS axis (34). With the increase in plasma ACE2, an increase in Ang-(1-7) was also expected. However, no difference was observed in plasma Ang-(1-7) levels in CKD patients and controls. Clinical and experimental studies revealed an increase in the concentration of serum ACE2, but with a parallel reduction in the expression of ACE2 in the kidney^35^^,^ 36. This finding was observed in the setting of diabetes associated to kidney disease. In addition, no difference in Ang-(1-7) concentrations was observed in these studies^37^. A possible explanation for the absence of increase in Ang-(1-7) levels is that the elevation of ACE2 concentration may not be associated with the enhancement in its activity. Therefore, the conversion of Ang II into Ang-(1-7) was not increased^37^. It should be mentioned, however, that plasma levels of Ang II were not measured in the present study.

Another possible explanation for the enhancement of plasma ACE2 levels in patients with CKD is the need to protect the kidneys from deleterious effects of Ang II. Thus, ACE2 may exert renoprotective effects. This possibility is further supported by the correlation between lower GFR values with lower ACE1/ACE2 ratios. 

The correlation analysis of the variables also showed that patients with higher ACR values had higher ACE1 levels (p<0.01), suggesting that the albumin excretion in the urine was accompanied by the increased in ACE1 expression, which is closely linked to the Ang II production that, in turn, produced kidney injury.

As described above, our results showed that decreased kidney function can lead to significant changes in the physiology of the renin angiotensin system in its different aspects. We suggest that increased levels of ACE2 may occur as a way to counterbalance the deleterious effects of ACE1 and angiotensin II on kidney function. Albuminuria that can be observed from the increase in ACR is strongly indicative of kidney glomerular injury with increased glomerular permeability possibly due to microvascular injury inside or in the vicinity of Bowman’s capsule; these findings concomitant with the increase in ACE levels 1 suggest a possible effect of Ang II in this process, but such evidence needs further investigation since in the present study Ang II dosage was not performed. 

The renin-angiotensin system plays an important role in regulating glomerular filtration. A decrease in kidney plasma flow is followed by an increase in the resistance of the glomerular efferent arteriole, so that perfusion pressure and glomerular filtration remain constant. In addition, if the decrease in kidney plasma flow is more pronounced, vasodilating prostaglandins, whose synthesis is stimulated by Ang II, dilate the afferent arterioles, which support glomerular filtration^38^. Like RAAS, other vasoactive molecules may also be involved in this process, which suggests the need for investigation of other molecular mechanisms that may be involved in the progression of kidney injury.

It is worth mentioning that this study has some limitations. First, the reduced number of participants may have limited study power and reduced number of variables collected. In addition, CKD patients were significantly older than the control group. It is known that the prevalence of CKD increases with advancing age, mainly due to cellular senescence, which culminates in reduction of the number of nephrons, and alteration in RAAS activity and vasoactive response ^(39, 40)^. Thus, age can be an important confounding factor that was not taken into account in the analysis performed. The study was based on the single determination of biological markers and not on repeated measurements over time.

In conclusion, our results show that patients with CKD had greater classic RAAS axis activity with compensatory response of the alternative axis, mostly by means of enhancement of plasma ACE2 levels. In addition, CKD patients had higher CCL2 plasma levels than controls. Understanding the effects of RAS and chemokines at the onset and during progression of CKD is very important, since this comprehension has the potential to reveal new prognostic markers and alternative therapeutic targets. However, despite the great advance in knowledge about pathophysiological mechanisms that relate the inflammatory response and RAAS to CKD, many aspects still need to be elucidated and further studies are necessary for this purpose.
